# Vascularized Chin Fat Flap for Mandibular Reconstruction in Stable Scleroderma En Coup De Sabre: A Case Report and Review of Surgical Considerations

**DOI:** 10.7759/cureus.82741

**Published:** 2025-04-21

**Authors:** Yuki Odagiri, Naoki Matsuura, Edward H Ntege, Reiko Asato, Yusuke Shimizu

**Affiliations:** 1 Plastic and Reconstructive Surgery, University of the Ryukyus Hospital, Ginowan, JPN

**Keywords:** autologous reconstruction, chin fat flap, facial atrophy, localised scleroderma, mandibular reconstruction, scleroderma en coup de sabre

## Abstract

Scleroderma en coup de sabre (ECDS) is a rare form of linear scleroderma that typically causes progressive atrophy on the face, which can affect both appearance and function over time. Although various reconstructive strategies exist, the use of a vascularized chin fat flap (CFF) for mandibular soft tissue restoration has not been previously reported. We describe a 61-year-old woman with stable ECDS and anterior mandibular depression. After disease stabilization with methotrexate, a pedicled fat flap was harvested from the submental region and inset via a single incision while preserving its native vascular supply. At six months, the patient exhibited sustained volume, restored facial symmetry, excellent scar quality, and no complications. The CFF combines vascular reliability, anatomical compatibility, and minimal invasiveness. Its ability to achieve natural, long-lasting results in a single stage, potentially under local anesthesia, makes it a promising alternative to conventional methods such as fat grafting, fillers, or implants in ECDS-related facial atrophy.

## Introduction

Scleroderma en coup de sabre (ECDS) is a rare subtype of linear scleroderma characterized by linear, atrophic depressions affecting the face or scalp, typically along Blaschko’s lines [1]. With an estimated prevalence of 0.4-2.7 per 100,000 individuals, ECDS predominantly affects females (approximately 3:1 female-to-male ratio) and typically presents during childhood or adolescence, although adult-onset cases occur [2-4]. ECDS is thought to result from a localized autoimmune process that induces inflammatory fibrosis and microvascular dysfunction, leading to progressive atrophy of the skin, subcutaneous tissue, and occasionally bone [1,5].

Although not life-threatening, ECDS can cause progressive atrophy of the skin, subcutaneous fat, and underlying bone, resulting in facial asymmetry and psychosocial distress. The condition typically progresses through an inflammatory phase lasting two to five years, followed by persistent residual deformities [6]. Up to 64% of patients develop osseous involvement, complicating reconstruction [5].

Medical therapy, particularly methotrexate, is the primary treatment to halt progression, demonstrating high efficacy when initiated early [7,8]. A minimum disease-free interval of six to 12 months is recommended before surgical intervention [9,10].

Reconstructive options for correcting soft tissue deficits in ECDS include autologous fat grafting, hyaluronic acid (HA) fillers, dermal-fat grafts, botulinum toxin, alloplastic implants, tissue expanders, and free flaps [8-12]. However, challenges persist due to unpredictable volume retention, multiple procedural requirements, and donor-site morbidity [5,8,9]. Reconstructive efforts in this population are further complicated by poor tissue pliability, compromised vascularity, increased fibrosis, and the potential risk of disease reactivation triggered by surgical manipulation [5,6]. The inherently thin and tethered overlying skin also complicates the achievement of natural contours and symmetry with conventional techniques [5].

Given these limitations, this report introduces the vascularized chin fat flap (CFF) as a novel, anatomically compatible, and minimally invasive reconstructive option for lower facial volume restoration in stable ECDS. Here, we describe the surgical approach and evaluate its position within current reconstructive strategies.

## Case presentation

A 61-year-old woman presented with a longstanding, progressive linear depression extending from her forehead to the left anterior mandible. She had not received prior treatment and was diagnosed with ECDS at the age of 60. Following one year of methotrexate therapy (10 mg/week), the disease stabilized clinically, and she was subsequently referred to our Department of Plastic and Reconstructive Surgery at the University of the Ryukyus Hospital for correction of mandibular contour.

**Figure 1:**
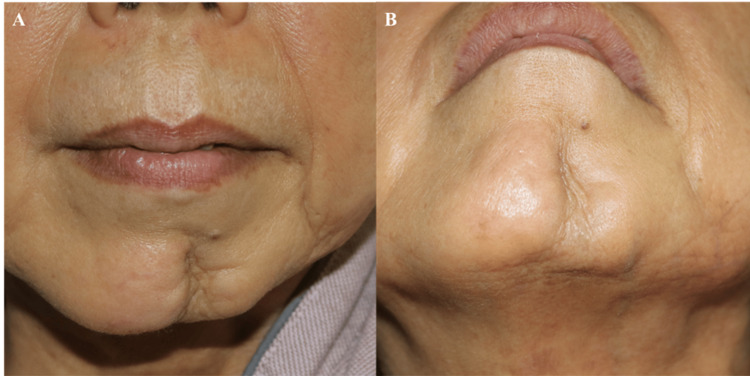
Preoperative views (A) Frontal view showing a depressed lesion on the left anterior mandible. (B) Oblique view demonstrating a linear depression toward the submandibular region.

Clinical examination revealed a sharply demarcated atrophic depression measuring approximately 8 cm in length and 1-2 cm in width, with a depth ranging from 3 to 8 mm. Digital analysis estimated an affected surface area of approximately 14 cm². Quantitative analysis of the CT scan data demonstrated a 32% soft tissue volume deficit on the affected side compared to the contralateral side. Although CT scan is not traditionally the first-line modality for superficial soft tissue assessment, it allowed reliable volumetric comparison of subcutaneous tissue depth in this case. This objective measurement was instrumental in determining the required dimensions for the planned reconstruction. While dedicated virtual surgical planning software was not utilized, standardized photographic analysis and CT-based volumetric comparison guided our flap design. The overlying skin appeared thin and tethered (Figure 1A, 1B).

**Figure 2:**
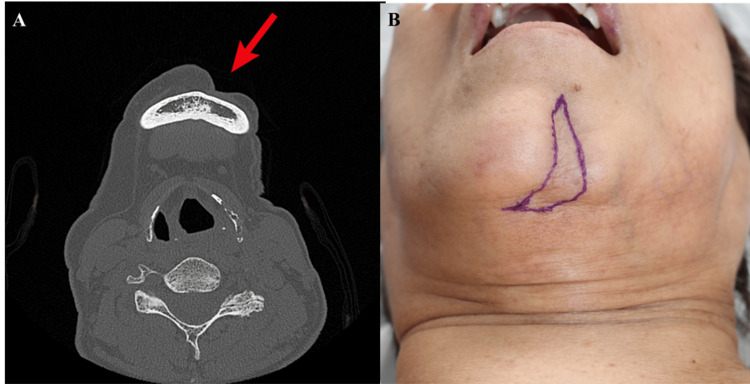
Preoperative imaging and planning (A) Axial CT showing soft tissue atrophy without bony involvement. (B) Skin marking, outlining scar excision, and flap design.

CT imaging confirmed soft tissue atrophy without underlying bony involvement (Figure 2A).

Based on the characteristic linear depression along Blaschko’s lines, confirmatory CT findings of isolated soft tissue atrophy, and clinical stabilization after one year of methotrexate therapy, the diagnosis of ECDS was firmly established. Differential diagnoses methodically excluded included Parry-Romberg syndrome (PRS, due to lack of deeper tissue and neurological involvement), traumatic or postsurgical scarring (ruled out by clinical history and lesion progression), linear morphea without craniofacial involvement (dismissed based on strictly facial localization), post-radiation atrophy (excluded by absence of radiation history), and hemifacial microsomia (eliminated given the clearly acquired and progressive presentation). This comprehensive diagnostic evaluation confirmed stable ECDS, thus providing a solid foundation for the subsequent reconstructive approach.

Preoperative markings were made to guide scar excision and flap design (Figure 2B).

**Figure 3:**
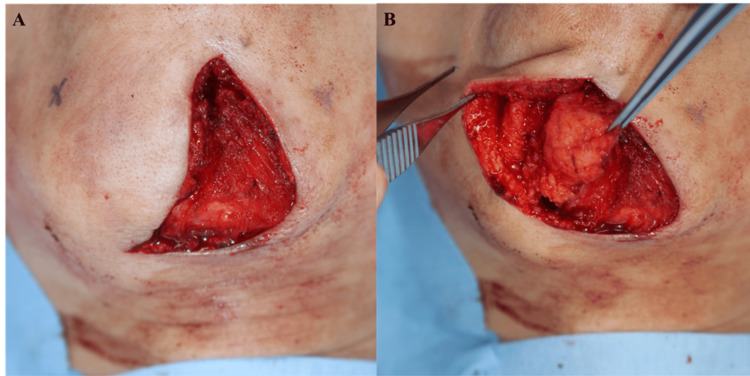
Intraoperative flap harvest (A) Scar excision and exposure of atrophic tissue. (B) Elevation of the CFF with preserved lateral pedicle. CFF, chin fat flap

Under general anesthesia (GA), a 7.2 cm incision was made along the mentolabial fold to excise the scar and release adhesions (Figure 3A). Given the moderate volume deficit, local tissue availability, and patient preference for minimal morbidity, a pedicled fat flap measuring 5.3 cm × 3.2 cm × 1.8 cm (approximately 30.5 cm³) was harvested from the right submental region through the same incision. Dissection was performed in a submuscular plane, carefully preserving the mentalis and depressor muscles. The vascular pedicle, approximately 1.2 cm wide, included branches from the facial, submental, and mental arteries (Figure 3B).

**Figure 4:**
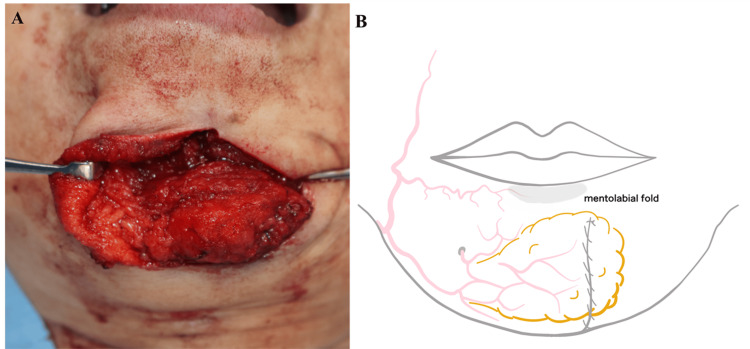
Flap insetting and design (A) Rotation and insetting of the flap into the defect. (B) Flap design aligned with the mentolabial groove.

The flap was rotated superomedially approximately 70° and secured to the periosteum using five 4-0 PDS sutures (Figure 4A). The flap contour was aligned with the mentolabial groove to ensure natural aesthetic restoration (Figure 4B).

**Figure 5:**
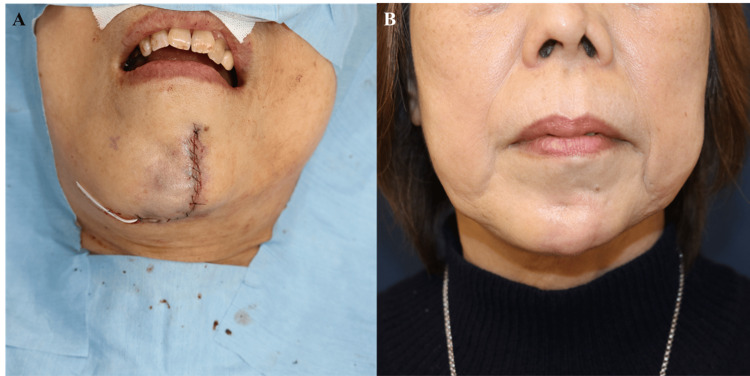
Wound closure and early result (A) Layered closure over the drain without tension. (B) Six-month postoperative frontal view showing contour restoration.

Closure was performed in layers using 5-0 Vicryl and 6-0 nylon sutures. A closed-suction drain placed intraoperatively was removed on postoperative day 2, with a total drainage of 22 mL (Figure 5A).

Prophylactic cefazolin (1 g IV) was administered intraoperatively to minimize infection risk, followed by oral cephalexin (500 mg four times daily for five days). The patient was advised to limit strenuous activity and excessive facial movement for two weeks. Sutures were removed on postoperative day 7, and gentle massage was initiated at three weeks.

**Figure 6:**
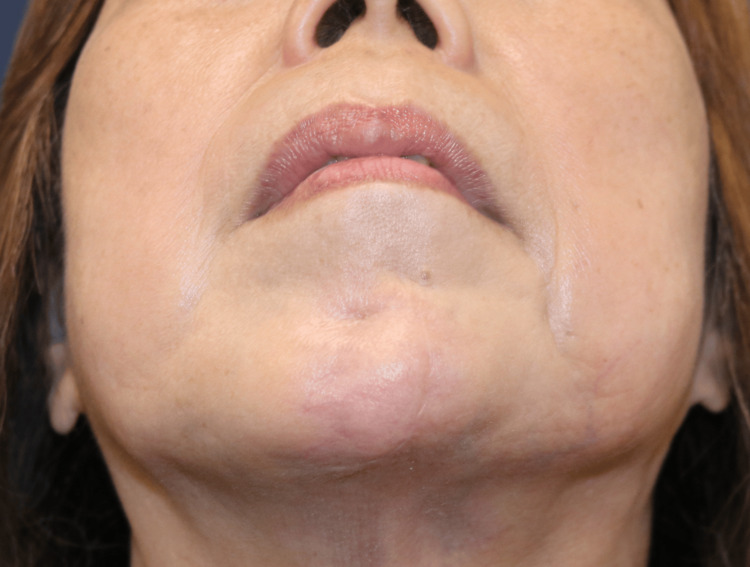
Six-month postoperative oblique view confirming volume maintenance and symmetry

At six months postoperatively, the flap remained volumetrically stable. Caliper measurements demonstrated correction within 1 mm of the contralateral side, and three-dimensional photographic analysis confirmed a 91% restoration of the preoperative volume deficit. The patient reported high satisfaction, rating her outcome as 9/10 on a visual analogue scale. The Patient and Observer Scar Assessment Scale (POSAS) yielded excellent scar quality scores of 12/60 (patient assessment) and 9/60 (observer assessment). No complications were observed (Figure 5B, Figure 6). Given the limited existing data on long-term outcomes of CFF reconstruction in ECDS, annual follow-up visits were scheduled to assess sustained flap viability and aesthetic stability.

## Discussion

This case demonstrates the feasibility and favorable outcomes achievable through the use of a vascularized CFF for mandibular contour restoration in a patient with stable ECDS. Although various reconstructive methods have been described to address soft tissue atrophy in ECDS, each carries trade-offs related to invasiveness, durability, and predictability. To our knowledge, this is the first report describing the application of a pedicled CFF specifically for this indication.

Autologous fat grafting remains the most commonly employed technique due to its simplicity, repeatability, and tissue compatibility. However, unpredictable graft resorption often necessitates multiple procedures to achieve satisfactory symmetry [8,9]. Dermal fillers, such as HA, represent minimally invasive alternatives offering immediate aesthetic results; however, their temporary nature and risk of foreign-body granulomatous reactions limit their long-term utility [10,11]. Botulinum toxin can effectively address dynamic asymmetry but provides no volumetric restoration [5].

More invasive methods, including tissue expansion, free flaps, and bone grafting, can adequately correct larger or more complex deformities but are technically demanding and carry significant risks of morbidity [13,14]. Alloplastic implants and bone cement offer durable structural solutions but pose substantial risks of infection, extrusion, and aesthetic mismatch [12]. A recent review of 39 ECDS patients reported that autologous fat grafting was utilized in 41% of cases, surgical reconstruction in 18%, and implants in 10% [5]. This discussion integrates established methods with recent clinical reports, such as patient-level outcomes detailed by Skorochod et al. [6] and the combined fat graft and demineralized bone matrix series presented by Menkü Özdemir et al. [8], thus positioning the CFF within contemporary reconstructive approaches. A comparative summary of these techniques is outlined in Table 1.

**Table 1:** Comparison of surgical options for mandibular volume restoration in ECDS CFF, chin fat flap; ECDS, en coup de sabre; HA, hyaluronic acid; LA, local anesthesia

Method	Advantages	Disadvantages	Reference(s)
Autologous fat grafting	Minimally invasive; repeatable; improves skin pliability	Unpredictable resorption; may require multiple sessions; inconsistent volume retention	[8,9]
HA filler	Office-based procedure; reversible; readily available	Temporary; risk of granuloma or foreign body reaction; unsuitable for atrophic skin	[10,11]
Botulinum toxin	Minimally invasive; useful for dynamic asymmetry	Temporary effect; no volumetric correction; possible adverse reactions	[5]
Alloplastic implants and bone cement	Durable; volume stable; effective in moderate defects	Risk of infection, extrusion, and foreign body reaction; poor tissue texture match	[12]
Tissue expander	Provides sufficient soft tissue volume; allows staged reconstruction	Requires two-stage surgery; risk of infection; patient discomfort	[13]
Free flaps/bone grafts	Suitable for large or complex defects; permanent correction	Highly invasive; requires surgical expertise; donor site morbidity	[14]
CFF	Autologous; vascularized; natural texture; single-stage, potentially under LA	Limited volume; unsuitable for distant or large defects; anatomical constraints	Present study

The CFF occupies a unique intermediate position, combining the advantages of minimal invasiveness and durability. As a pedicled, vascularized flap harvested from adjacent adipose tissue, it provides reliable perfusion, anatomical compatibility, and minimal donor-site morbidity, all achievable through a single-stage procedure. The concept of utilizing local submental fat tissue for chin augmentation was first described by Robertson in 1965, who developed a double CFF with preserved blood supply for chin enhancement [15]. Later, Hamra expanded on techniques for addressing the aging chin through mentalis-periosteal flap advancement [16]. Our application adapts these foundational concepts specifically for facial volume restoration in ECDS, preserving the key principle of vascular integrity while tailoring the approach to address the unique challenges of localized scleroderma.

**Figure 7:**
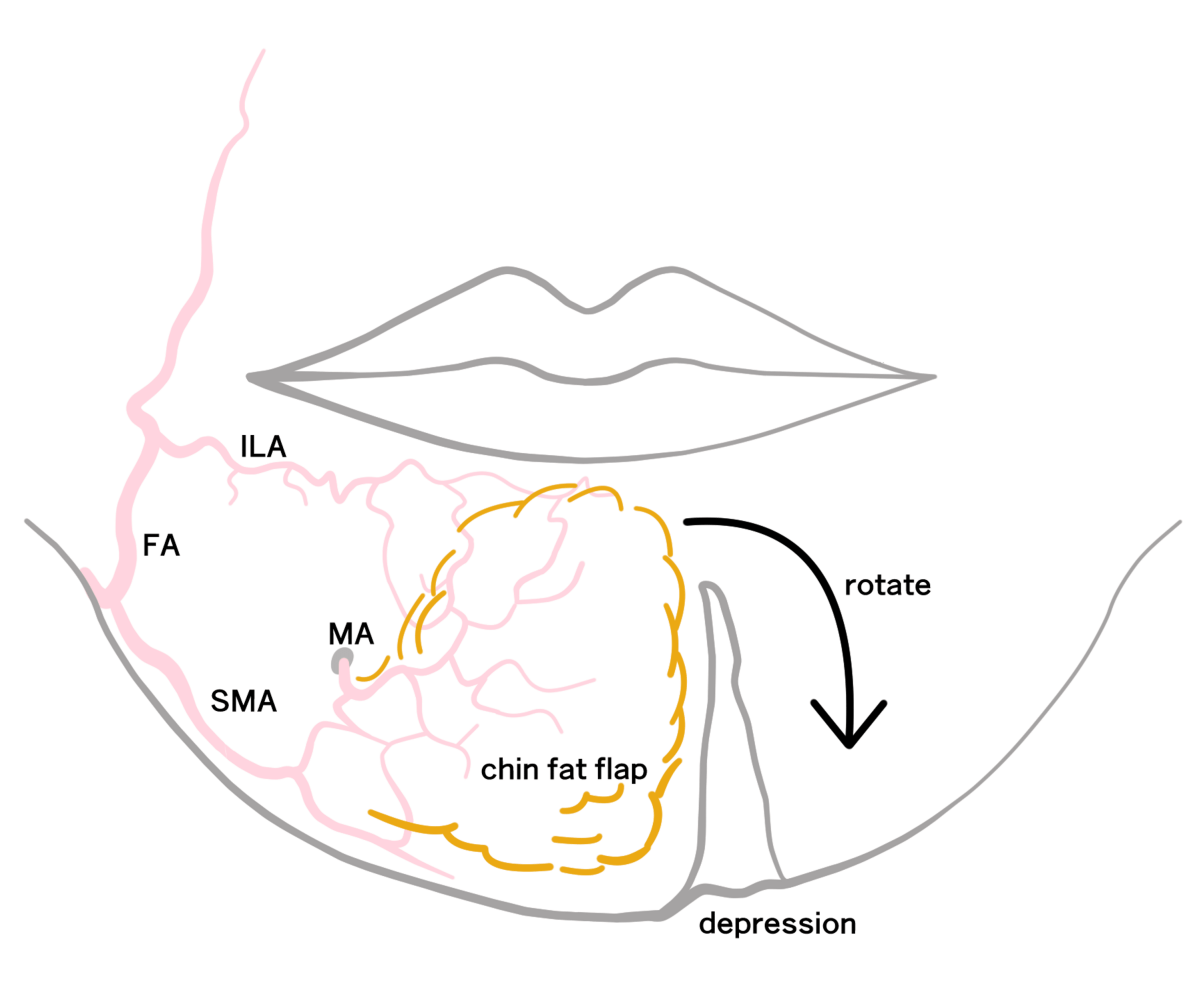
Schematic illustration of flap perfusion anatomy The submental region receives blood supply from the FA and its branches: SMA, ILA, and MA. The lateral pedicle preserves vascular continuity during CFF elevation. CFF, chin fat flap; FA, facial artery; ILA, inferior labial artery; MA, mental artery; SMA, submental artery

The submental region is richly vascularized by branches of the facial artery, specifically the submental and mental arteries (Figure 7) [17]. In our patient, the flap was elevated within a submuscular plane, carefully preserving facial musculature. It was inset without tension and securely anchored to the periosteum. At six months postoperatively, the flap demonstrated stable volume retention with 91% restoration confirmed by three-dimensional photographic analysis, and patient satisfaction was excellent.

Compared to fat grafting, the inherent vascularity of the CFF may reduce resorption and enhance long-term volume retention. Unlike free flap procedures, the CFF eliminates the need for microsurgical expertise and significantly shortens operative time. Moreover, aligning the flap with the natural mentolabial groove resulted in inconspicuous scarring and a harmonious aesthetic transition, validated by favorable POSAS scores. Although additional contouring procedures such as autologous fat grafting, HA, or dermal fillers were not indicated at this stage due to the excellent initial results and sustained volume retention, these adjunctive treatments were discussed with the patient preoperatively and at follow-up visits as options should residual or future contour discrepancies emerge. The reliable vascularization of the CFF provided distinct advantages over fat grafting alone, particularly in maintaining stable volume and reducing the need for repeated interventions.

While this is a single case report and therefore has limited statistical generalizability, we incorporated objective and semi-objective assessments where appropriate. These included caliper-based thickness comparison, validated scar scoring using POSAS, and a patient-reported visual analogue scale rating for satisfaction. Future studies may benefit from incorporating additional tools such as magnetic resonance imaging for volumetric monitoring and the Facial Appearance-Related Psychosocial Impact Questionnaire (FACE-Q) to standardize patient-reported aesthetic and quality-of-life outcomes.

PRS, which shares overlapping clinical characteristics with ECDS, has been similarly addressed using local volume augmentation techniques [18]. Despite differences in etiology, both conditions may benefit substantially from autologous, vascularized soft tissue reconstruction.

While the procedure was performed under GA in this study, the limited operative field and minimal dissection suggest it could alternatively be performed under local anesthesia (LA) with appropriate nerve blocks, potentially offering reduced recovery time and hospital stay for suitable candidates.

This approach is best suited for moderate-volume defects in patients with adequate submental adiposity. However, it may be inappropriate for cases with extensive or multi-zone facial atrophy. Short-term results were favorable, but the absence of longer-term follow-up and the limitation of this single-case report restrict the generalizability of the findings. Further studies involving larger cohorts and prolonged observation periods are necessary to validate the technique’s durability, refine patient selection criteria, and assess long-term aesthetic and functional outcomes.

## Conclusions

This case demonstrates the clinical utility of the vascularized CFF as an effective and underutilized option for mandibular soft tissue reconstruction in stable ECDS. The single-incision technique enables reliable volume restoration using adjacent, vascularized tissue, minimizing morbidity and eliminating the need for implants or microsurgery. Our patient achieved over 90% volumetric restoration, excellent scar quality, and high satisfaction at six months.

The CFF is best suited for moderate-volume defects in patients with adequate submental fat and may be feasible under LA. While early outcomes are promising, further studies with larger cohorts and longer follow-up are necessary to confirm durability and define optimal indications. This report supports the CFF as a valuable addition to the reconstructive armamentarium for facial atrophy in ECDS.
